# Memory training through Chinese classics recitation for individuals with subjective cognitive decline: study protocol for a pilot study

**DOI:** 10.3389/fnagi.2026.1830299

**Published:** 2026-07-20

**Authors:** Tz-Syuan Su, Kuan-Pin Su, Po-Ren Hsueh, Zi-Lun Lai, Chun-Ming Chen, Yi-Chun Lai, Chien-Sin Chen, Xing Nian, Ya-Wen Chang, Sheng-Ta Tsai

**Affiliations:** 1 School of Medicine, College of Medicine, Kaohsiung Medical University, Kaohsiung, Taiwan; 2 Office of Research and Development, Asia University, Taichung, Taiwan; 3 An-Nan Hospital, China Medical University, Tainan, Taiwan; 4 School of Medicine, College of Medicine, China Medical University, Taichung, Taiwan; 5 Department of Laboratory Medicine, China Medical University Hospital, China Medical University, Taichung, Taiwan; 6 Department of Medical Imaging, China Medical University Hospital, Taichung, Taiwan; 7 Neuroscience and Brain Disease Center, College of Medicine, China Medical University, Taichung, Taiwan; 8 Department of Psychiatry, China Medical University Hospital, Taichung, Taiwan; 9 Department of Dermatology and Immunology, University of Pittsburgh, Pittsburgh, PA, United States; 10 Yellow Flower Meditation Academy, New York, NY, United States; 11 School of Nursing, College of Nursing, Asia University, Taichung, Taiwan; 12 Department of Neurology, China Medical University Hospital, Taichung, Taiwan; 13 Neural Engineering and Precision Surgery Laboratories, Mayo Clinic, Rochester, MN, United States

**Keywords:** Chinese classics, memory training, pilot study, recitation, subjective cognitive decline

## Abstract

**Background:**

Subjective cognitive decline (SCD) is a preclinical condition associated with an increased risk of dementia and Alzheimer’s disease, with no consensus regarding effective therapeutic interventions to date. As the global burden of aging-related neurodegenerative diseases continues to rise, early intervention has become increasingly critical. Cognitive training has been proposed as a promising behavioral intervention; however, few studies have integrated multimodal assessments to elucidate its underlying neurobiological and psychological mechanisms, particularly within the educational system that shapes cognitive reserve and learning strategies across the lifespan. This pilot study aims to estimate effect sizes for future definitive trials, assess the feasibility and acceptability of the intervention and multimodal evaluations, and generate hypotheses regarding the relationship between recitation practice and clinical biomarkers.

**Methods:**

A total of 60 individuals with subjective cognitive decline will be enrolled in a randomized, assessor-blinded, controlled pilot study. Participants will be randomly assigned to either an intervention group or a non-active control group prior to baseline assessment. The intervention group will undergo a six-month structured recitation training program. Both groups will undergo short-term follow-up after the intervention, and long-term follow-up annually post-intervention. Multimodal assessments will include neuropsychological testing, functional magnetic resonance imaging (fMRI), electroencephalography (EEG), blood biomarker profiling, gut microbiota analysis, and fecal metabolomics.

**Discussion:**

This study protocol outlines a randomized, assessor-blinded, controlled pilot study designed to advance understanding of the feasibility of Chinese Classics recitation training on both physiological and psychological outcomes in individuals with subjective cognitive decline. The findings are expected to provide mechanistic insights into non-pharmacological interventions and to address the current underrepresentation of Asian populations in neuroscience research.

**Clinical trial registration:**

We registered this randomized controlled trial on ClinicalTrials.gov on March 11, 2026, under the identifier NCT07463391.

## 1 Introduction

Dementia, a progressive neurodegenerative condition strongly associated with aging, imposes substantial social, economic, and familial burdens worldwide. Its prevalence is projected to continue rising, posing significant challenges to global public health systems (Liu et al., 2025). In Asia, the increase has been particularly pronounced: from 1990 to 2021, the incidence, prevalence, and mortality of Alzheimer’s disease (AD) and related dementias increased by 244.73, 250.44, and 297.34%, respectively (Guo et al., 2025). These alarming trends underscore the urgent need for early identification and intervention strategies targeting preclinical stages of cognitive decline.

Subjective cognitive decline (SCD) was formally conceptualized in 2014 to describe a self-reported experience of cognitive deterioration in individuals who perform normally in objective and standardized neuropsychological tests (Jessen et al., 2020). The trajectories of SCD are heterogeneous, ranging from reversible or stable forms to progressive phenotypes that evolve into mild cognitive impairment (MCI) and eventually dementia (Jessen et al., 2020). A meta-analysis reported that approximately 14% of individuals with SCD developed dementia over a four-year follow-up period (Mitchell et al., 2014). Despite its clinical significance as a potential preclinical stage of neurodegeneration, no pharmacological treatments are currently approved specifically for SCD (Smid et al., 2022).

For patients with established dementia, current treatment approaches broadly fall into biological and behavioral domains. Pharmacological management commonly includes cholinesterase inhibitors, such as rivastigmine, and N-methyl-D-aspartate receptor antagonists, such as memantine (Byeon et al., 2025). In 2021, the U. S. Food and Drug Administration approved aducanumab (Aduhelm), an amyloid-targeting monoclonal antibody, marking a milestone in disease-modifying therapeutic development for Alzheimer’s disease. Subsequently, lecanemab demonstrated greater clinical efficacy in reducing cognitive decline and cerebral amyloid burden compared to placebo (van Dyck et al., 2023). However, treatment with amyloid-targeting antibodies has been associated with amyloid-related imaging abnormalities, including cerebral edema and microhemorrhage, particularly among individuals who are apolipoprotein E ε4 homozygotes (van Dyck et al., 2023). These safety concerns highlight the continued importance of developing non-pharmacological interventions with favorable risk–benefit profiles, especially for individuals at earlier stages such as SCD.

Cognitive training represents one of the most extensively studied behavioral interventions for SCD. A meta-analysis of 16 randomized controlled trials reported that cognitive training interventions were associated with improvements in individuals with SCD, with no significant statistical heterogeneity detected across studies (Bhome et al., 2018). Notably, statistically significant improvements were primarily observed when interventions were compared with non-active control groups that lacked structured cognitive stimulation (Bhome et al., 2018). This finding suggests that external cognitive engagement may be a critical factor in driving measurable benefits. Despite accumulating evidence supporting non-pharmacological approaches, research tailored to Asian populations remains limited. Among the trials included in the aforementioned meta-analysis, only one study specifically focused on individuals whose primary language was Chinese (Tsai et al., 2008). This disparity highlights a shortage of culturally and linguistically adapted cognitive intervention research for Chinese-speaking populations. In contrast to other neurological assessments, such as muscle strength evaluation, the assessment of higher cortical functions is inherently culture-dependent (Rosselli et al., 2022). For example, in the Boston Naming Test, items such as igloo and harp may be relatively unfamiliar to Chinese participants, whereas the abacus—often considered one of the more challenging items for American participants—is widely recognized among many Chinese individuals (Li et al., 2022).

More recent investigations have further demonstrated that non-pharmacological interventions, including cognitive training, transcranial direct current stimulation, balance exercises, music therapy, and mindfulness-based therapies, are associated with overall positive effects in individuals with SCD (Yu X.-H et al., 2024). However, few studies have incorporated multimodal biological assessments to elucidate the mechanistic pathways underlying observed behavioral improvements.

Classical recitation constitutes a multimodal cognitive activity that concurrently engages phonological, articulatory, semantic, and auditory self-monitoring circuits. Evidence from functional MRI confirms that recitation activates the primary motor cortex, supplementary motor areas, Broca’s area, Wernicke’s area, and the superior temporal gyrus — regions critically susceptible to age-related decline in SCD (Ranjan and Singh, 2025). Sustained verbal training in older adults has been demonstrated to enhance frontotemporal and hippocampal functional connectivity, with articulatory exercises specifically reinforcing the phonological retrieval networks most affected by aging (Yang et al., 2022). At the network level, structured verbal-attentional practice fortifies intrinsic connectivity within the default mode network — particularly between the hippocampus and posteromedial cortex — which is the network most selectively disrupted in preclinical Alzheimer’s disease (Sevinc et al., 2021).

The proposed pathway from recitation to gut microbiota modulation is mediated through autonomic nervous system regulation. Focused, rhythmic vocalization reduces hypothalamic–pituitary–adrenal (HPA) axis reactivity and enhances vagal tone, thereby attenuating stress-driven gut dysbiosis (Jabbari Shiadeh et al., 2025). Sustained psychological stress has been shown to alter neurotransmitter release and disrupt intestinal epithelial homeostasis by blocking protective vagal nerve barrier effects, shifting microbial composition toward pro-inflammatory profiles (Ningthoujam et al., 2021). Conversely, mind–body practices that enhance vagal tone are associated with healthier gut microbiota diversity and reduced pro-inflammatory bacteria. The mechanistic link between gut microbiota and brain plasticity operates primarily through microbial metabolites, as emerging evidence highlights the critical role of the gut-brain axis, a complex bidirectional network involving neural, endocrine, immune, and metabolic pathways (Tsai et al., 2025). Within this framework, gut microbiota has been shown to influence blood–brain barrier integrity, neuroplasticity, and cognitive function through the production of short-chain fatty acids, modulation of neurotransmitter precursors, and regulation of systemic inflammation (Agirman and Hsiao, 2021; Zhang et al., 2023). For instance, gut bacteria produce butyrate, acetate, and propionate, which cross the blood–brain barrier and upregulate the CREB/BDNF signaling pathway, promoting neuronal repair, memory consolidation, and synaptic plasticity, while suppressing neuroinflammation via inhibition of MAPK and NF-κB (Qian et al., 2022). In parallel, microbial tryptophan metabolism yields indole derivatives and kynurenine pathway intermediates that modulate serotonin synthesis and glutamatergic signaling — both critical for hippocampal neuroplasticity (Connell et al., 2022). Recent advances in whole-blood metabolomics have further identified disease-associated metabolic signatures in individuals with dementia (Teruya et al., 2021), underscoring the potential of metabolic profiling as a tool for early detection. These findings collectively suggest that alterations in the gut-brain axis may occur in the preclinical stages of cognitive decline. However, a direct causal link between classical recitation and modulation of the gut microbiome has not yet been established in any interventional trial, and whether cognitive training prevents or delays cognitive decline by protecting gut microbiota composition and metabolic profiles, particularly in individuals with SCD, has not been systematically investigated. Therefore, decoding how cognitive interventions modulate the degenerative interactions of gut-brain axis will generate significant impacts on preventive medicine.

Cognitive training programs often incorporate culturally tailored elements to enhance acceptability and engagement among participants. In practice, training materials delivered in English may be ineffective for individuals who do not understand the language. However, culturally matched cognitive training programs remain scarce in Chinese-speaking societies despite rapid demographic aging and the growing burden of dementia. Therefore, this study with Chinese cognitive training materials investigates whether this culture-match, structured cognitive training is related to outcomes in individuals with SCD and examines potential neurobiological and systemic correlates of any observed effects, such as blood inflammation marker (cortisol). Specifically, we propose to evaluate the impact of Chinese Classics recitation training through comprehensive multimodal assessments, including neuropsychological testing, functional magnetic resonance imaging (fMRI) (Rajah and D'Esposito, 2005; Anthony and Lin, 2018), electroencephalography (EEG) (Yu W. Y. et al., 2024), blood biomarker profiling (Teruya et al., 2021), and gut microbiota (Kuijer and Steenbergen, 2023) and fecal metabolomic analyses (Zhao et al., 2025). By integrating behavioral intervention with advanced neuroimaging and biomarker methodologies within a culturally grounded educational framework, this study seeks to address current gaps in the literature and provide population-specific evidence relevant to Chinese-speaking communities. Such an approach may contribute to the development of precision-oriented, low-risk intervention strategies for early cognitive decline in aging populations. We will primarily aim to evaluate the feasibility and acceptability of classical recitation training in older adults with subjective cognitive decline. As a secondary objective, we will investigate whether recitation training correlates with changes in neuroimaging markers associated with brain plasticity. Additionally, exploratory objectives include characterizing metabolomic and gut microbiota profiles and examining their potential associations with cognitive and neuroimaging outcomes. These exploratory analyses are designed to generate preliminary data and inform future hypothesis-driven research exploring potential pathways linking recitation training, gut-brain interactions, and neuroplasticity, representing a novel mechanistic framework.

## 2 Methods and analysis

### 2.1 Aims, study design, and settings

This study aims to estimate preliminary effect sizes to inform sample sizes for future definitive trials, evaluate the feasibility and acceptability of memorization practice and multimodal assessments, and generate mechanistic hypotheses about the relationship among recitation practice, neuroimaging biomarkers, and gut-brain axis markers, particularly for individuals with SCD. By analyzing changes in fMRI, neuropsychological assessments, and physiological biomarkers, including blood sampling, plasma metabolomic profiling, and fecal microbiota and metabolomic analyses, we seek to elucidate both the short-term practicability of structured recitation training and its underlying neurobiological mechanisms in SCD. Regarding long-term implications, our database will primarily consist of Chinese-speaking Asian participants. The longitudinal follow-up data will enable a more comprehensive understanding of the progression of SCD within this specific population, thereby addressing an underrepresented demographic in current SCD research.

The study is designed as a randomized, assessor-blinded, controlled pilot study. A stratified block randomization sequence will be generated by an independent statistician using computer-generated random numbers, with stratification by age and sex to ensure balanced group allocation. Block sizes will be randomly varied to prevent predicting upcoming assignments. Allocation concealment will be ensured using the Sequentially Numbered, Opaque, Sealed Envelopes method; envelopes will be prepared and safeguarded by personnel not involved in recruitment, eligibility assessment, or intervention delivery. After eligibility assessment and informed consent are completed, a designated research assistant — otherwise uninvolved in outcome assessment — will open the envelope to reveal group assignment. Prior to allocation disclosure, all research personnel will remain blinded to the randomization sequence. Given that the intervention is behavioral in nature, complete blinding of participants and interventionists is not feasible. However, several measures will be implemented to minimize performance bias. Participants will be instructed not to discuss their group assignment with outcome assessors. Interventionists will have no role in outcome measurement or data analysis. Outcome assessors — including neuropsychological evaluators (psychologists) and radiologic technologists — will be formally blinded to group allocation throughout the trial and will be kept separate from the intervention team. To maintain blinding integrity, assessors will be reminded of their blinded status at each assessment visit, and any unblinding events will be documented and reported. Data analysts will similarly remain blinded to group assignment until the primary analysis is complete. The inherent risk of performance bias arising from participant awareness of group assignment will be mitigated by standardizing intervention protocols, using objective outcome measures where possible, and monitoring for differential dropout between groups.

**Figure 1:**
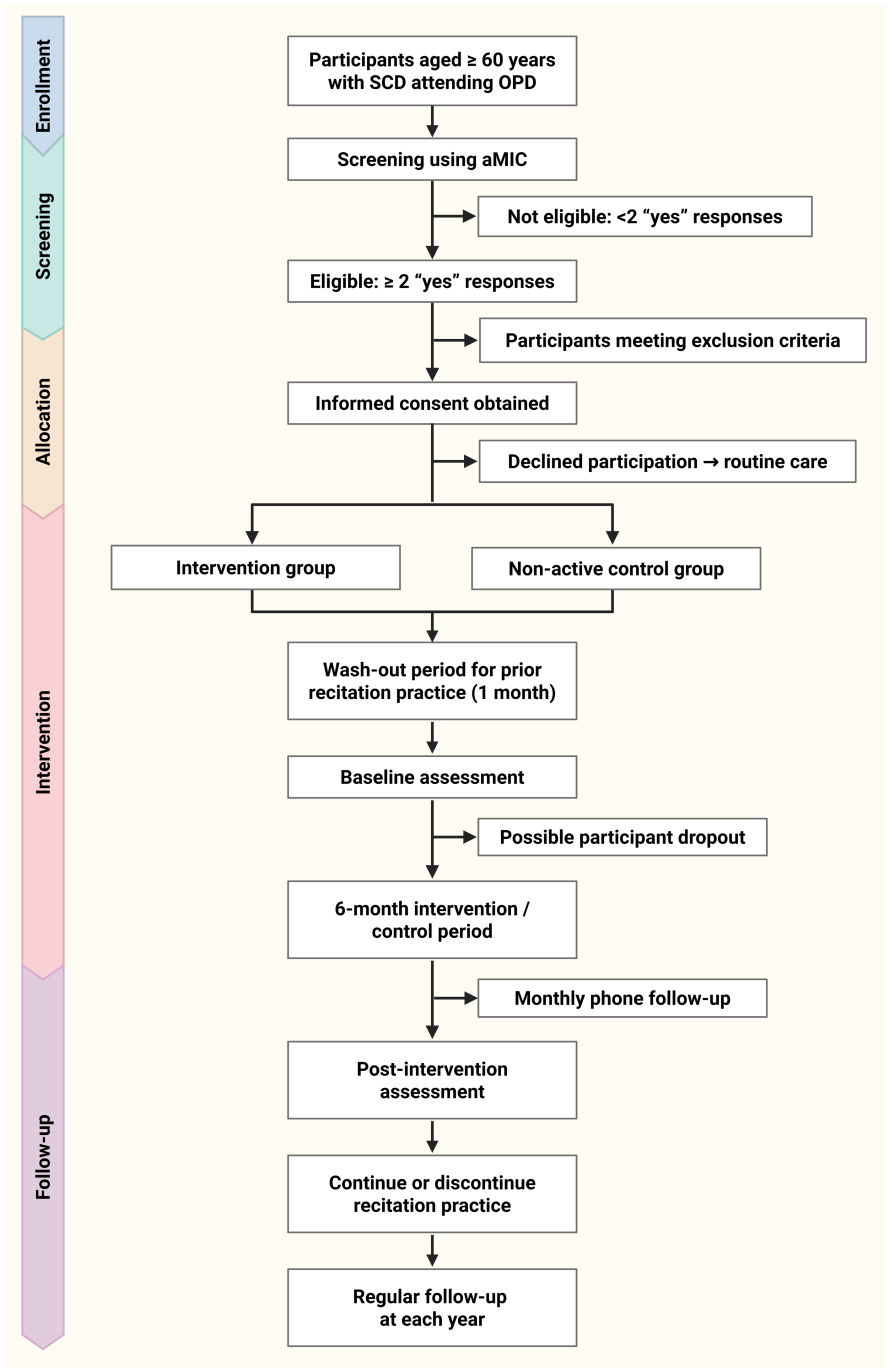
Workflow of the randomized controlled trial. This flowchart illustrates the recruitment, screening, and enrollment process for participants. aMIC, abbreviated Memory Inventory for the Chinese; OPD, outpatient department; SCD, subjective cognitive decline.

Participants will be randomly assigned to either an intervention group or a non-active control group (Figure 1). This design decision is informed by a meta-analysis conducted by Bhome et al., which reported that cognitive training interventions in SCD demonstrated statistically significant improvements primarily when compared with non-active control conditions. In contrast, comparisons with active control groups receiving alternative forms of cognitive stimulation (e.g., watching educational films) did not yield statistically significant differences (Bhome et al., 2018). The absence of an active control group, a cognitive placebo condition, a social interaction control group, and a Hawthorne effect control group represents a recognized limitation of the present design. However, the decision to use a non-active control was made to maximize detection sensitivity in this pilot study, consistent with prior literature (Bhome et al., 2018). Future definitive trials should incorporate an active control condition matched for time, social contact, and participant engagement to permit more rigorous causal interference.

The intervention group will undergo structured memorization training through recitation of Chinese Classics for 6 months. The intervention will be delivered by trained research assistants in weekly 60-min sessions, supplemented by approximately 10 min of individual daily practice over the six-month period. The control group will not receive memorization training during this period. To ensure reproducibility, a detailed intervention manual has been developed specifying recitation materials, progression criteria, and instructor guidance. All instructors will complete a standardized training program prior to the commencement of the study, encompassing both the theoretical rationale for the intervention and practical delivery skills. Instructor competency will be formally assessed before authorization to deliver sessions. As this study is conducted at a single center — China Medical University Hospital — inter-center standardization is not applicable to the current protocol; however, the intervention manual will be designed to support multi-site replication in future trials.

Intervention fidelity will be monitored through the following method. Daily home practice will be tracked via automated logs within the mobile application, capturing login frequency, session duration, and task completion rates. Adherence will be operationally defined as completion of at least 80% of the designated training, including at a minimum of 80% of scheduled training sessions and the fulfillment of at least 80% of the assigned daily practice throughout the six-month duration. Participants falling below this threshold at any monthly review will be contacted by the research team to identify and address barriers to engagement.

Following completion of the initial six-month intervention phase, participants will be given the option to voluntarily continue recitation practice. The selection of a six-month intervention period is informed by the MEMO+ (Méthode d’Entrainement pour Mémoire Optimale) protocol developed by Sylvie Belleville and colleagues (Bier et al., 2015). Later studies from the same research team have demonstrated that participants receiving structured cognitive training exhibited significant improvements in memory recall performance, with effects persisting for at least 6 months post-intervention (Belleville et al., 2018). Furthermore, long-term follow-up over 5 years revealed that individuals who had undergone cognitive training experienced a slower decline in delayed memory performance and Montreal Cognitive Assessment (MoCA) scores compared with control groups (Belleville et al., 2024). These findings underscore the sustained benefits and potential long-term impact of continuous cognitive training.

Additional support for the selected intervention duration is derived from studies examining other forms of cognitively engaging activities. For example, interventions involving structured calligraphy practice have demonstrated that extending training duration over a six-month period leads to significant alterations in functional brain connectivity (Lee et al., 2024). Specifically, increased connectivity has been observed between the medial prefrontal cortex (mPFC) and right lateral temporal cortex (LTC), mPFC and right inferior parietal lobe (IPL), left hippocampal formation (HF) and right LTC, as well as between the left HF and right IPL (Lee et al., 2024). These findings suggest that prolonged engagement (6 months) in cognitively demanding tasks may induce measurable neuro-modulatory effects at the network level.

Taken together, a 6-month intervention period represents a balanced design choice: it is sufficiently long to allow for detectable behavioral and neurobiological changes, yet not so prolonged as to compromise participant adherence. Accordingly, outcome assessments will be conducted at baseline (time of recruitment), immediately after the six-month intervention, and at one-year and two-year follow-ups, with annual assessments thereafter until participants are no longer able to complete evaluations. The study will be conducted at China Medical University Hospital, primarily within the outpatient clinics of the Departments of Neurology or Psychiatry.

### 2.2 Participants

Participants aged 60 years or older will be recruited, consistent with internationally recognized SCD-Initiative consensus criteria proposed by Jessen et al. (2014). Eligible individuals must report a subjective perception of cognitive decline persisting for more than 6 months and not attributable to a specific acute event, in accordance with criteria described by Molinuevo et al. (2017). SCD will be formally confirmed based on the SCD-Initiative criteria (Jessen et al., 2014), incorporating both self-report and a structured clinical interview. All participants must provide written informed consent and meet the inclusion and exclusion criteria detailed below.

Increasing evidence suggests that therapeutic interventions targeting earlier stages of neurodegenerative disease may yield greater clinical benefit compared with interventions initiated at more advanced stages. For example, disease-modifying therapies such as donanemab have been investigated primarily in individuals with early symptomatic Alzheimer’s disease (MMSE score of 20–28), reflecting the growing emphasis on early intervention (Mintun et al., 2021). Similarly, cognitive training strategies are recommended when implemented at earlier phases of cognitive decline (He et al., 2023). Based on this evidence, we selected individuals with SCD, who represent a preclinical or very early stage along the cognitive decline continuum, rather than patients with MCI. This approach allows evaluation of whether structured cognitive training may exert measurable effects prior to substantial neurodegeneration, thereby maximizing the potential sensitivity for detecting both behavioral and biological changes.

As part of the screening process, we will administer the “Abbreviated Memory Inventory for the Chinese” developed by Chinese University of Hong Kong (Lam et al., 2005). The instrument consists of five questions assessing subjective memory concerns.

1. Forgetting where things are placed?
2. Unable to recall the names of good friends?
3. Unable to follow and recall conversation?
4. Subjective memory problems?
5. Consider own memory to be worse than others of a similar age?

Prior evidence indicates that an increasing frequency of subjective memory complaints is associated with higher Clinical Dementia Rating (CDR) scores. Specifically, individuals endorsing more than three out of five items on the Abbreviated Memory Inventory for the Chinese are more likely to be associated with MCI or Alzheimer’s disease. Notably, previous findings also demonstrated that subjective memory complaints are highly prevalent among cognitively normal individuals, with approximately 70.2% reporting subjective memory problems and 63.8% reporting difficulty recalling the location of objects (Lam et al., 2005). These observations are consistent with our clinical experience and highlight the need to distinguish early-stage SCD from more advanced cognitive impairment.

To balance sensitivity for detecting SCD while minimizing the inclusion of participants with undiagnosed MCI, we adopted a threshold of “at least two” positive responses on the five-item inventory as an eligibility criterion. This cutoff allows inclusion of individuals with meaningful subjective cognitive concerns while reducing the likelihood of enrolling participants with more advanced cognitive impairment. To exclude early MCI, all participants will undergo objective cognitive assessment using standardized instruments (e.g., MoCA score ≥26) (Sun R. et al., 2023). Individuals performing 1.5 SD or more below age- and education-adjusted norms on any cognitive domain will be excluded. Given that depressive and anxiety symptoms can independently contribute to subjective cognitive complaints, all participants will be screened using the Geriatric Depression Scale (GDS) and/or the Generalized Anxiety Disorder scale (GAD-7). Individuals meeting clinical thresholds for significant depression or anxiety will be excluded, or alternatively, these scores will be included as covariates in the statistical analysis.

Exclusion criteria:

- Objective cognitive impairment defined as ≥1.5 standard deviations below standardized cognitive testing (e.g., MMSE) (Belleville et al., 2023).
- Diagnosis of MCI (e.g., MoCA score <26) (Sun R. et al., 2023).
- Clinical Dementia Rating (CDR) ≥ 0.5 (Molinuevo et al., 2017).
- Diagnosis of major depressive disorder (Molinuevo et al., 2017).
- Beck Anxiety Inventory score ≥16 (means moderate to severe anxiety).
- Other medical or neurological conditions that could explain cognitive decline (e.g., vascular dementia, traumatic brain injury).
- Claustrophobia.
- Contraindications of MRI (e.g., non-MRI-compatible cardiac pacemaker or devices implanted within the past 3 months).

### 2.3 Sample size

Our sample size calculation is based on selecting functional MRI changes as an exploratory mechanistic outcome measure, given their higher sensitivity than neuropsychological tests at the preclinical stage in SCD patients (Viviano and Damoiseaux, 2020). This approach differs from many previous cognitive intervention studies that have used neuropsychological test scores as primary outcomes. The target sample size is informed by prior neuroimaging studies in individuals with SCD, where changes in functional MRI parameters served as the primary outcomes (Bhome et al., 2018), while also considering feasibility constraints (Belleville et al., 2023). Accordingly, we plan to recruit 60 participants, with 30 individuals allocated to each group. To account for an anticipated attrition rate of approximately 20%, based on dropout rates reported in comparable longitudinal cognitive intervention studies in older adults (Cheng et al., 2024; Fernandez-Gamez et al., 2026), we will initially enroll 72 participants (36 per group), with the expectation that at least 60 will complete the study.

If neuropsychological test scores were used as the primary outcome, a substantially larger sample size would be required. An *a priori* power analysis was conducted using G*Power version 3.1.9.4 (Heinrich Heine University, Düsseldorf, Germany), based on a two-group comparison of continuous outcomes within the t-test family (“Means: Difference between two independent means”). Equal group allocation was assumed (N₂/N₁ = 1), and a one-tailed test was selected given the directional hypothesis that the intervention would improve outcomes. The anticipated standardized effect size was set at Cohen’s d = 0.18, derived from prior meta-analytic evidence indicating a small-to-moderate effect consistent with neuroimaging-based outcomes in SCD populations (Bhome et al., 2018). With a Type I error rate (*α*) of 0.05 and statistical power (1 − *β*) of 0.80, the estimated required sample size would be 383 participants per group (total *N* = 766; df = 764; actual power = 0.8006). Given the extensive multimodal assessments and longitudinal design of the present study, recruiting a sample of this magnitude is not feasible. Therefore, this study is designed as an exploratory, hypothesis-generating trial with a smaller sample size, aiming to provide preliminary effect estimates and inform future large-scale confirmatory studies.

The present study acknowledges that the planned sample of 60 participants is underpowered for the neuropsychological and other multimodal secondary outcomes. The adequacy of statistical power for these secondary outcomes — which include cognitive test scores, subjective cognitive measures, and additional neuroimaging parameters — was not the basis for the sample size determination and should be interpreted with caution. Effect sizes from secondary outcomes in this pilot trial will inform power calculations for future definitive trials.

### 2.4 Interventions

The memorization intervention consists of structured recitation of Chinese Classics for a minimum of 5 min per day. The selection of Chinese Classics will be determined collaboratively by the research team and participants. Various memorization strategies will be introduced, including intensive repetition techniques and visualization strategies. Participants may begin by reading the designated text aloud for at least 5 min daily before progressing to full memorization. The research team recommends beginning with the classical text “Di Zi Gui,” translated as Standards for Being a Good Student. To facilitate adherence and standardization, a mobile application currently under development will assist participants in memorization training, provide access to digital literature materials, and record daily recitation duration.

After six consecutive months of training, participants in the intervention group will undergo a standardized recitation assessment. The primary performance metric will be the number of correctly recited Chinese characters within a fixed time frame. For example, Di Zi Gui contains 1,080 Chinese characters. Participants will be required to recite the entire text within 11 min, with no more than eight omitted or incorrectly recited characters. Participants who meet these criteria will be classified as having passed the assessment. The recitation assessment will be conducted by qualified judges affiliated with the Bliss & Wisdom Foundation of Culture and Education, who are capable of reciting the complete text from memory. During evaluation, judges will reference the original text while participants perform their recitation to ensure objective scoring accuracy.

### 2.5 Outcome measurement

As described in the Sample Size section, functional MRI serves as an exploratory mechanistic outcome measure, detecting changes in task-related brain activation. The unit of measurement will be the percentage change in whole-brain blood-oxygen-level-dependent signal (%). APOE genotyping will be offered as an optional ancillary component of the study and used for exploratory analyses to investigate potential associations between genetic risk and intervention outcomes.

All other outcomes are defined as secondary outcomes, including:

1. changes in resting-state EEG spectral activity;
2. performance changes across 11 neuropsychological tests;
3. APOE genotype distribution;
4. circulating inflammatory and stress biomarkers (IL-6, TNF-*α*, high-sensitivity C-reactive protein, and cortisol);
5. gut microbiota diversity (Shannon index);
6. gut microbiota taxonomic composition; and
7. fecal metabolomic profiles related to the gut–brain axis.

Detailed methodologies for each outcome measure are described in the following sections.

#### 2.5.1 Clinical evaluations and laboratory testing

A structured research questionnaire will be administered to collect comprehensive baseline information, including age, biological sex, years of education, comorbidities, medication history, family history of neurodegenerative diseases, smoking and betel nut use, dietary preferences, eating habits, coffee or tea consumption (Zhang et al., 2026), bowel movement patterns, and prior experience with memorization training (e.g., structured memorization of Chinese Classics for more than 3 months). The questionnaire may be completed independently by participants or administered by trained research personnel. In addition to questionnaire-based assessments, participants will undergo basic physical examinations and detailed neurological evaluations.

#### 2.5.2 Brain functional assessments

Electroencephalography (EEG) and non-contrast structural MRI will be performed prior to enrollment to exclude structural or vascular lesions that may account for cognitive decline, such as significant white matter hyperintensity (total Fazekas score ≥3; Fazekas et al., 1987) or internal carotid artery (ICA) occlusion. These exclusion criteria are informed by two lines of evidence. First, prior studies on cognitive reserve and white matter hyperintensities have demonstrated that increased white matter burden is associated with cognitive decline independent of Alzheimer’s disease pathology, potentially confounding the assessment of disease-specific effects (Lin et al., 2025). Second, ICA occlusion has been identified as a significant vascular risk factor for cognitive impairment and dementia (Baradaran et al., 2021). Therefore, excluding participants with substantial white matter hyperintensity or ICA occlusion helps minimize vascular contributions to cognitive decline and ensures a more homogeneous study population.

##### 2.5.2.1 EEG acquisition and processing

Resting-state EEG recordings will be acquired using a standard 21-electrode montage positioned according to the international 10–20 system. Electrode impedance will be maintained below 10 kΩ, with a minimum sampling rate of 256 Hz. Recordings will be conducted in a quiet, dimly lit environment, with participants seated comfortably and instructed to remain awake with their eyes closed. EEG data preprocessing will be performed using Python 3 and the MNE-Python software package (Yu W. Y. et al., 2024). The preprocessing pipeline will include re-referencing, resampling to 250 Hz, and band-pass filtering between 0.1 and 41 Hz. Cleaned EEG signals will then be segmented into non-overlapping 2-s epochs for subsequent analysis.

##### 2.5.2.2 MRI acquisition protocol

MRI data will be acquired using a 3.0 Tesla scanner (GE Signa Architect, Milwaukee, WI, USA) equipped with a 40-channel head coil. Both structural and functional imaging data will be collected. High-resolution three-dimensional T1-weighted images will be obtained using either spoiled gradient recalled echo (3D-SPGR) or inversion recovery-prepared SPGR sequences, with isotropic spatial resolution of 1 × 1 × 1 mm^3^. These images will be used for anatomical localization, co-registration, spatial normalization, and region-of-interest (ROI) definition. Functional MRI (fMRI) data will be acquired using T2-weighted echo-planar imaging sequences with whole-brain coverage. Imaging parameters will include: repetition time (TR) = 2000 ms, echo time (TE) = 35–60 ms (depending on optimization), flip angle = 90°, matrix size = 64 × 64, field of view ≈ 224 × 224 mm^2^, slice thickness = 4.0–4.4 mm with a 0.4 mm interslice gap, and voxel size ≈ 3.5–3.6 × 3.5–3.6 × 4.4 mm^3^. A total of 32 axial slices will be acquired in interleaved order.

##### 2.5.2.3 Experimental paradigm and imaging schedule

Resting-state and task-based fMRI will be conducted at baseline and after the six-month intervention period. During resting-state scans, participants will be instructed to remain awake with their eyes closed and avoid structured cognitive activity. Task-based fMRI will employ a block-design paradigm. During task blocks, participants will be instructed to silently recite previously memorized Chinese Classics or mentally retrieve learned material, serving as the cognitive task. In addition, amyloid positron emission tomography (PET) imaging may be performed at participants’ own expense at any point during the study. Longitudinal follow-up PET imaging (e.g., 5 years post-intervention) will enable intra-individual comparisons of amyloid deposition trajectories and long-term disease progression.

##### 2.5.2.4 fMRI preprocessing

All fMRI preprocessing and statistical analyses will be conducted using Statistical Parametric Mapping (SPM; version 8 or later) implemented in MATLAB (MathWorks, Natick, MA, USA). Preprocessing will follow a standardized pipeline:

- Slice Timing Correction: Adjustment for temporal differences in slice acquisition, performed prior to realignment for interleaved sequences.
- Realignment (Motion Correction): Correction for head motion using rigid-body transformation; six motion parameters will be estimated and included as nuisance regressors.
- Co-registration: Alignment of functional images to individual T1-weighted structural images for accurate anatomical localization.
- Spatial Normalization: Transformation of structural images into Montreal Neurological Institute space, with parameters applied to functional images.
- Spatial Smoothing: Application of a Gaussian kernel (full width at half maximum [FWHM] = 8 mm) to improve signal-to-noise ratio and meet statistical assumptions.

##### 2.5.2.5 Statistical analysis

Statistical analyses will be conducted within the framework of the general linear model (GLM). For the primary clinical outcomes (e.g., subjective cognitive measures, neuropsychological test scores), a linear mixed-effects model will be used to handle the repeated-measures structure of the data throughout baseline, post-intervention, and follow-ups, with time, group, and group × time interaction as fixed effects, and subject as a random effect. This approach naturally accommodates missing data under the missing-at-random (MAR) assumption without requiring complete cases. The primary analysis will follow the intention-to-treat principle, including all randomized participants regardless of adherence or dropout, with missing values handled using multiple imputation based on baseline characteristics and available follow-up data. A per-protocol sensitivity analysis will also be conducted, restricted to participants who complete at least 80% of the intervention sessions. Covariates to be included in adjusted models will comprise age, sex, years of education, and baseline cognitive scores, selected *a priori* for their known associations with cognitive outcomes in SCD populations.

For the neuroimaging analyses, task-related regressors will be convolved with the canonical hemodynamic response function, and motion parameters will be included as nuisance covariates. A high-pass filter of 128 s will be applied to remove low-frequency drift. At the single-subject level, voxel-wise GLM analyses will be performed to identify task-related activation patterns. Contrasts of interest (e.g., recitation versus resting baseline) will be evaluated using directional T-contrasts. At the group level, individual contrast maps will be entered into second-level random-effects models. Directional two-sample T-tests will be used for between-group comparisons, and conditions with similar activation patterns may be combined to enhance statistical power. Statistical significance will be set at *p* < 0.05, corrected for multiple comparisons using the false discovery rate (FDR) at the voxel level. For exploratory analyses, an uncorrected threshold of *p* < 0.001 may be applied. Extracted effect sizes from predefined ROIs will be further analyzed using Statistical Product and Service Solutions (SPSS). One-way analysis of variance will be used to assess condition-related differences, while Spearman’s rank-order correlation will evaluate associations between neural activation and clinical variables (e.g., years of education and Cognitive Reserve Index questionnaire (CRI-q) scores). Statistical significance will be defined as *p* < 0.05.

#### 2.5.3 Neuropsychological assessments

Neuropsychological evaluations will assess six principal cognitive domains:

1. Memory.
2. Temporal and spatial orientation.
3. Executive function.
4. Visuospatial ability.
5. Attention and calculation.
6. Language.

These domains will be evaluated using standardized instruments, including:

- Mini-Mental State Examination (MMSE).
- Montreal Cognitive Assessment (MoCA).
- Rey Auditory Verbal Learning Test.
- Stroop Color and Word Test.
- Digit Span Test (Nasiri et al., 2023).
- Boston Naming Test - Short Form (total score = 15).

#### 2.5.4 Plasma and fecal metabolome analysis by LC–MS/MS

For fecal metabolite extraction, stool samples were homogenized with 3 mL of ethanol, followed by centrifugation, and 100 μL of the resulting supernatant was collected and vacuum-dried. For plasma samples, protein precipitation was performed by adding methanol to a 20 μL aliquot at a 1:4 (v/v) ratio; the supernatant was collected and vacuum-dried. Both dried extracts were reconstituted in 20 μL of 5% (v/v) aqueous methanol containing 250 fmol isotopically labeled internal standards, of which 5 μL was injected for LC–MS/MS analysis. Chromatographic separation was performed on an Atlantis T3 column (3 μm, 100 Å, 2.1 × 150 mm, Waters) at 35 °C and 250 μL/min, using 0.1% formic acid in water (mobile phase A) and acetonitrile (mobile phase B), with a gradient of 1% B for 1 min, ramping to 40% B over 14 min, 80% B over 3 min, held for 3 min, and re-equilibrated at 1% B for 5 min (total run time: 25 min). Mass spectrometric detection was performed on a QTRAP 6500 + system (AB Sciex) in both negative and positive ion modes; in negative mode, CUR, CAD, and IS were set at 25 psi, 12, and −4,500 V, respectively, and in positive mode at 25 psi, 10, and +5,500 V, with source temperature, GS1, and GS2 maintained at 500 °C, 50 psi, and 60 psi for both modes. Targeted quantification was conducted in MRM mode, with precursor ion, product ion, and retention time individually predefined for each analyte; peak areas were integrated using Analyst software (AB Sciex).

#### 2.5.5 Gut microbiota profiling by full-length 16S rRNA gene sequencing

Full-length 16S rRNA gene sequencing will be performed using the PacBio Revio platform, enabling species-level taxonomic resolution for precise identification of microbial strains capable of producing neuroactive metabolites. PCR amplification will be conducted using universal primers 27F and 1492R, followed by SMRTbell library preparation and sequencing, yielding approximately 10,000 high-quality circular consensus sequencing (CCS) reads (Q30) per sample. Bioinformatic processing will be performed using QIIME2, with taxonomic classification based on the SILVA database. Downstream analyses will include alpha and beta diversity indices (e.g., Shannon index), taxonomic annotation, and differential abundance analysis between study groups using DESeq2. DNA extraction and library preparation procedures will follow those described by Tsai et al. (2025).

### 2.6 Statistical methods

The primary efficacy analysis will be conducted using generalized linear mixed-effects models to analyze repeated measures data, with adjustment for relevant covariates. Microbiome analyses will be performed using DESeq2 to identify differentially abundant taxa and linear discriminant analysis effect size to detect potential microbial biomarkers. Metabolomic data will be analyzed using partial least squares discriminant analysis, and pathway enrichment analysis will be conducted using the MetaboAnalyst platform. Multi-omics integration will be performed using Spearman correlation-based network analysis and causal mediation analysis to investigate regulatory mechanisms along the gut-brain axis. Multiple comparisons will be corrected using FDR, with a threshold of *q* < 0.05. All statistical analyses will be conducted using R (version 4.4.0; R Core Team, 2024) and SPSS (version 29.0; IBM Corp., Armonk, NY, USA), with statistical significance set at *p* < 0.05.

Given the inherent complexity and multi-layered nature of multi-omics data, if the aforementioned methods are insufficient for comprehensive analysis, we will collaborate with the institutional Artificial Intelligence Center affiliated in our hospital to implement advanced analytical approaches. These may include machine learning, network-based modeling, and partial least squares regression, among others, to construct predictive models that characterize causal or associative relationships between biological markers and clinical outcomes (e.g., cognitive function and depression). The predictive performance of different models will be systematically compared.

### 2.7 Trial administration and governance

#### 2.7.1 Sponsorship and oversight

The trial is sponsored by the China Medical University Hospital. The sponsors and funders have no role in the study design, data collection, management, analysis, or interpretation. A Steering Committee, composed of senior researchers in neuroscience and clinical neurology, oversees trial progress. An independent Data Management Team at China Medical University Hospital handles data auditing. A formal Data Monitoring Committee was not deemed necessary due to the non-invasive nature of the behavioral intervention in this trial. No formal interim efficacy analysis was planned, and early stopping for efficacy was not considered applicable. However, safety is monitored by the Steering Committee, whereas trial conduct is monitored quarterly by the Research Ethics Committee.

#### 2.7.2 Participant retention and care

Participants may withdraw at any time upon request. The intervention may be discontinued if a participant develops a medical condition that prevents daily recitation. Participants are permitted to continue their usual medical care but are requested to avoid starting other new structured cognitive training programs during the six-month study period. To promote retention, research assistants maintain monthly contact via phone calls. This trial involves a non-invasive behavioral intervention; no specific post-trial care is required. However, any adverse events, such as MRI-related discomfort, will be assessed during follow-up visits and reported to the Research Ethics Committee, ensuring participants receive medical care at China Medical University Hospital.

#### 2.7.3 Blinding and unblinding

While the control group is non-active, outcome assessors and data analysts remain strictly blinded to group assignments. Unblinding is only permissible in the event of a medical emergency in which the group assignment is critical to the participant’s safety.

#### 2.7.4 Data management and missing data

Data entry is performed by trained personnel with range checks to promote quality. Missing data will be handled using multiple imputation or the Last Observation Carried Forward method. Sensitivity analyses will be conducted to assess the robustness of the primary findings with respect to participant adherence levels.

## 3 Discussion

### 3.1 Theoretical framework and mechanistic rationale

Among non-pharmacological cognitive interventions for SCD, computerized cognitive training (CCT) — the most extensively studied approach — yields a small but statistically significant overall effect on untrained cognitive outcomes in healthy older adults (Hedges’ *g* = 0.18, 95% CI 0.14 to 0.23), with benefits primarily in processing speed, working memory, and non-verbal memory, though with considerable heterogeneity across trials (*I*^2^ = 58%) and wide prediction intervals spanning negligible to moderate effects (−0.36 to 0.73) (Lampit et al., 2020). Exercise-based interventions represent a second major modality; a network meta-analysis of exercise interventions specifically in SCD populations found that, while exercise improved multiple cognitive domains, it remained unclear which type of exercise was most effective, and no single modality demonstrated consistent superiority (Chen et al., 2024). Combined cognitive-physical interventions produce a small advantage over single-domain cognitive training on executive functions, but achieve similar effects on memory and global cognition (Rieker et al., 2022). Notably, a network meta-analysis of pharmacological and non-pharmacological interventions for SCD across 56 randomized controlled trials found that education-based programs ranked highest for improving both memory and global cognition — above CCT, physical exercise, and pharmacological approaches — suggesting that structured, content-rich verbal-cognitive engagement may be a particularly promising avenue (Roheger et al., 2021).

Despite growing evidence for the general benefit of cognitive interventions in aging, the magnitude and consistency of effects vary substantially across trials, limiting the conclusions that can be drawn from aggregate findings. A comprehensive systematic review of interventions specifically targeting SCD found that, while cognitive training yielded a small statistically significant improvement in objective performance overall (*g* = 0.13, 95% CI 0.01 to 0.25), effect sizes became non-significant when studies used active control groups (*g* = 0.02) or reported global cognitive outcomes (*g* = 0.06), indicating that published effects may largely reflect expectancy and practice artifacts rather than genuine cognitive benefit (Roheger et al., 2021). Heterogeneity of effects is attributable to multiple sources, including variability in SCD diagnostic criteria, differences in intervention dosage and delivery format, inconsistent outcome measurement across domains, and the absence of standardized follow-up periods. In addition, the methodological quality of published cognitive intervention trials in SCD and related preclinical populations remains a significant concern. The majority of trials suffer from one or more of the following limitations: inadequate sample sizes, short intervention durations with no long-term follow-up, outcomes measured only on trained tasks rather than ecologically valid measures, high or unclear risk of bias due to lack of assessor blinding, and failure to conduct intention-to-treat analyses (Roheger et al., 2021). Beyond sample size and reporting concerns, cognitive intervention trials face several systematic biases that may inflate apparent treatment effects. The most pervasive is performance bias from expectancy effects, in which participants believe they are receiving an active intervention and therefore may perform better on outcome assessments regardless of the intervention’s true efficacy. A systematic review of computerized cognitive training trials in healthy older adults found that only 47% included an active control condition, and only 21% measured participants’ expectations — meaning that the majority of published positive findings cannot rule out expectancy as a major contributor to the observed effects (Masurovsky, 2020). To address the gap limited by short follow-up periods, inadequate control of participant expectancy effects, and a predominant reliance on behavioral outcomes without accompanying biological measures in previous studies, this pilot study will incorporate long-term annual follow-ups, administer the credibility and expectancy questionnaire to monitor and statistically adjust for expectancy at baseline and post-intervention, and employ a multimodal assessment framework integrating cognitive, neuroimaging, metabolomic, and gut microbiota measures.

Epidemiological evidence has consistently identified modifiable lifestyle factors associated with reduced risk of cognitive decline and dementia. A landmark study published in *The Lancet Neurology* demonstrated that rich social networks, engagement in mental activities, and regular physical activity represent three major protective factors against cognitive decline, Alzheimer’s disease, and dementia in older adults. Among these, mental activities were particularly associated with preserved verbal intelligence and reduced rates of cognitive deterioration. Proposed mechanisms include the cognitive reserve hypothesis, the vascular hypothesis, and the stress hypothesis (Fratiglioni et al., 2004).

The cognitive reserve framework posits that lifelong intellectual enrichment enhances neural efficiency, capacity, and flexibility, thereby allowing individuals to better tolerate age-related neuropathology before clinical symptoms emerge. Substantial interindividual variability exists in cognitive aging trajectories; for example, some individuals in their eighties may demonstrate superior cognitive performance compared to middle-aged adults (Cabeza et al., 2018). A 20-year longitudinal study involving 3,777 older adults concluded that education was a key determinant of this variability, with higher educational attainment associated with delayed clinical manifestation of cognitive impairment by approximately 7 years (Amieva et al., 2014). These findings support the concepts of cognitive maintenance and reserve, suggesting that education and sustained mental engagement enhance neuroplasticity and increase resilience to neuropathological burden. Importantly, cognitive reserve is not static. It accumulates through education and occupational complexity during midlife but may decline in later life depending on physical health, psychosocial stress, and ongoing cognitive engagement (Cabeza et al., 2018). Structured cognitive training may therefore serve as a modifiable intervention to reinforce reserve mechanisms during aging. In the present study, fMRI will be employed to detect subtle alterations in neural activation patterns and network connectivity associated with recitation-based cognitive training.

Education does not only contribute to cognitive reserve but also modulates neural recruitment patterns during cognitive tasks. A study led by Sylvie Belleville demonstrated that individuals with higher educational attainment exhibited distinct activation patterns following cognitive training using the method of loci. Participants engaged in memory training for 1 h per day over three consecutive weeks. During memory retrieval tasks, individuals with higher education levels showed differential activation in the right middle and inferior temporal gyri (Brodmann areas 20, 21, and 37), regions associated with semantic processing and memory integration (Belleville et al., 2023). These findings suggest that education influences compensatory neural strategies and training-induced plasticity. Accordingly, years of education will be recorded in our cohort, and the CRI-q (Lin et al., 2025) will be administered to quantify reserve-related variables. Where sample size permits, subgroup analyses stratified by educational attainment will be conducted to examine differential intervention effects.

Beyond cognitive reserve mechanisms, the stress hypothesis provides an additional neurobiological framework. This model emphasizes dysregulation of the HPA axis. Chronic stress or maladaptive stress responses may lead to sustained glucocorticoid hypersecretion, downregulation of hippocampal glucocorticoid receptors, and impairment of the negative feedback loop regulating cortisol levels. Prolonged exposure to elevated glucocorticoids is associated with hippocampal neuronal loss and has been implicated in the pathogenesis of dementia (Fratiglioni et al., 2004). Through comprehensive biomarker profiling of blood samples, including metabolic and inflammatory markers, our study aims to explore whether structured memorization training may modulate systemic pathways related to stress regulation and neuroprotection.

Conceptually, Chinese Classics recitation departs from the dominant single-domain training paradigm underlying conventional cognitive training. Programs such as computerized working memory or attention training isolate one cognitive process and train it through repetitive, decontextualized trials, an approach associated with reliable near-transfer but inconsistent far-transfer (Owen et al., 2010; Simons et al., 2016). Recitation, by contrast, requires the concurrent integration of sustained attention, verbal learning, episodic memory retrieval, semantic comprehension, and executive control during continuous oral production — a structure more consistent with enrichment-type interventions linked to cognitive reserve, in which simultaneous multi-system engagement is hypothesized to support broader, distributed benefit rather than process-specific gains (Stern, 2009). This conceptual distinction extends to stimulus content and learning mechanisms. Where conventional tasks use abstract, content-free stimuli optimized for psychometric precision, recitation uses semantically dense, culturally meaningful text; under levels-of-processing accounts of memory, such meaningful material is expected to support deeper and more durable encoding than decontextualized information (Craik and Lockhart, 1972). The intervention also incorporates repeated oral retrieval of previously learned passages, engaging retrieval practice (testing effect) mechanisms associated with enhanced long-term retention (Roediger and Karpicke, 2006) — a feature largely absent from trial-based computerized training, which typically emphasizes recognition or reaction-based responding rather than self-generated recall.

Practically, the two approaches also differ in delivery. Conventional cognitive training is typically administered as discrete, algorithmically adaptive trials on an individual basis, with difficulty calibrated by software in response to real-time performance. Recitation is self-paced and continuous, often delivered in a group or instructor-guided setting, with dosage and repetition flexibly determined by the participant’s progress rather than by automated adjustment. This difference in implementation, combined with the use of culturally familiar material, gives recitation greater ecological validity, as it more closely resembles real-world language and memory processes than computerized drills and may support more sustained engagement. In addition to these conceptual and practical distinctions, the two approaches are also hypothesized to differ physiologically. Within the stress-axis framework, the rhythmic vocalization and breath regulation inherent in recitation are hypothesized to engage vagal and HPA-axis pathways, offering a candidate physiological mechanism — distinct from standard training paradigms — through which behavioral intervention could influence stress-related neuroplasticity and gut-brain signaling.

Collectively, the following theoretical models: cognitive reserve, neural compensation, and stress-axis modulation, provide the mechanistic foundation for the present trial. While multiple potential mechanisms may underlie the effects of classical recitation on cognitive function, our primary hypothesis is that classical recitation training will be associated with greater change in resting-state functional connectivity within the hippocampal-default mode network than controls, consistent with intervention-induced neuroplasticity in memory-relevant circuits. Our secondary hypothesis proposes that the stress-axis modulation pathway, whereby recitation-induced enhancement of vagal tone and attenuation of HPA axis reactivity diminish stress-related gut dysbiosis, consequently fostering the production of beneficial microbial metabolites and neuroplasticity. Exploratory hypotheses include associations between classical recitation and shifts in gut microbiota composition and in circulating metabolomic profiles. By integrating behavioral training with multimodal biological measurements, including neuroimaging and peripheral biomarker analyses, this study seeks to bridge cognitive neuroscience theory with measurable physiological outcomes in individuals with subjective cognitive decline.

### 3.2 Strengths and limitations

A major strength of this study lies in its focus on a Chinese-speaking population, which remains underrepresented in longitudinal cognitive training research. By integrating multimodal assessments and long-term follow-up, this study aims to provide novel insights into the trajectory of SCD and the potential disease-modifying effects of sustained cognitive training within this population.

Several limitations should be acknowledged. First, recruitment may be challenging, as individuals with SCD often present with mild symptoms and may not seek medical attention in outpatient settings. Second, the comprehensive pre- and post-intervention assessments are time-intensive and may reduce participant willingness and adherence. Third, participants may be aware of whether they are assigned to the intervention or control group because of the use of a non-active control group, which may introduce performance and expectancy biases. Additionally, the use of a mobile application for training may pose usability challenges for older adults, potentially contributing to dropout.

To mitigate these limitations, participants will be recruited from both Neurology and Psychiatry departments, as well as through collaborations with community activity centers to enhance outreach. To improve adherence, only individuals willing to complete study assessments will be enrolled, and the training platform will be optimized for elderly users (e.g., larger Chinese characters and simplified interface design). Adherence will be operationally delineated as the completion of no less than 80% of the designated intervention, specifically including attendance at a minimum of 80% of scheduled training sessions and the fulfillment of at least 80% of the assigned daily practice throughout the six-month duration. Adherence will be monitored continuously throughout the trial using automated session logs recorded in the mobile application, and any participant who falls below the threshold will be flagged for follow-up by the research team. Reasons for non-adherence and dropout will be systematically documented to inform the interpretation of findings and the design of future trials.

To address expectancy-related bias, the present study acknowledges that participants in the intervention group may attribute cognitive improvements to the mere receipt of a structured program rather than to its specific active components — a cognitive placebo effect that cannot be fully disentangled from true intervention effects in the absence of an active control condition. We therefore incorporate validated credibility and expectancy questionnaires administered at baseline and post-intervention to enable statistical adjustment for potential influences on outcomes. Participant expectancy will also be assessed at enrollment to characterize the degree to which anticipation of benefit may have influenced self-reported outcomes. Future definitive clinical trials should employ an active control condition that matches the intervention in time, attention, and participant engagement to better isolate the specific effects of the intervention and reduce the risk of biases.

The risk of cross-group contamination should also be acknowledged. As participants are recruited from overlapping settings — including outpatient departments and community centers — individuals assigned to the control group may become aware of the intervention content through social interaction with participants in the intervention group. This could attenuate between-group differences and lead to an underestimation of true intervention effects. To minimize this risk, participants will be reminded at enrollment and throughout the trial not to discuss study procedures with others. Future trials should consider cluster randomization or geographically separated recruitment sites to further reduce the risk of contamination.

The cultural and linguistic specificity of the intervention represents an additional limitation. The cognitive training program incorporates Traditional Chinese poetry recitation, a practice deeply embedded in Taiwanese cultural and educational traditions. The cognitive demands, memorability, and emotional engagement associated with this material may not translate equivalently to populations with different linguistic backgrounds or cultural relationships to classical literary forms. Accordingly, the generalizability of findings to non-Chinese-speaking populations may be limited. Adaptation and revalidation of the intervention content are required before international replication.

Given the modest sample size, statistical power may be limited. If interim analyses indicate insufficient statistical power, additional participants may be recruited. Conversely, if statistically significant results are observed earlier than anticipated, early termination of recruitment may be considered.

### 3.3 Anticipated outcomes

Prior neuroimaging research provides a strong theoretical basis for the expected neural changes in this study. A synthesis of 13 studies involving individuals aged over 60 years, including both cognitively healthy participants and those with MCI, demonstrates distinct functional activation patterns associated with cognitive reserve and compensatory mechanisms (Anthony and Lin, 2018). Specifically, medial temporal regions, such as the posterior cingulate cortex, parahippocampal gyrus, superior temporal gyrus, fusiform gyrus, medial temporal gyrus, superior temporal gyrus, and hippocampus, are consistently linked to cognitive reserve (Anthony and Lin, 2018). In contrast, frontal regions, including the medial, superior, and inferior frontal gyri, as well as the precentral gyrus, and the dorsal attention network are associated with compensatory processes (Anthony and Lin, 2018). Notably, a reduced signal ratio between temporal and frontal regions has been proposed as a marker of cognitive decline and neurodegeneration (Anthony and Lin, 2018).

Additional evidence from task-based fMRI studies indicates dynamic, stage-dependent neural changes. Increased activation of the left inferior prefrontal gyrus has been observed during memory encoding following cognitive training, whereas decreased activation in bilateral fronto-striatal regions has been reported during memory retrieval, suggesting improved neural efficiency (Belleville et al., 2023). Furthermore, education level appears to modulate neural activation patterns: individuals with lower educational attainment exhibit lower baseline activation in the right temporal lobe but demonstrate greater increases over time compared to those with higher education levels (Belleville et al., 2023). Complementary to fMRI, EEG has been established as a non-invasive and temporally sensitive biomarker for neurodegenerative processes. In Alzheimer’s disease, EEG findings typically include increased theta and delta power, decreased alpha and beta power (Baradaran et al., 2021), and disrupted functional connectivity across brain regions (Özbek et al., 2021). Similar findings involving 143 participants with 60 AD and 83 non-AD individuals have been shown in our previous study (Yu W. Y. et al., 2024).

Consistent with the hypothesis-generating nature of this pilot research, study outcomes are organized within a predefined inferential hierarchy. The primary outcome is designated as the principal efficacy endpoint and will serve as the main basis for evaluating intervention-related effects. The secondary outcome is pre-specified *a priori*; however, given the limited sample size typical of pilot studies, findings will be interpreted as supportive and hypothesis-generating rather than definitive. EEG, gut microbiota, and metabolomic measures are designated as exploratory outcomes. These measures are not powered for confirmatory inference and are intended primarily to generate preliminary effect-size estimates and identify candidate biomarkers for future adequately powered confirmatory trials.

Based on these findings, we anticipate that the primary outcome of this pilot trial is intervention-induced change in resting-state fMRI activation within the left medial temporal region, encompassing the hippocampus and parahippocampal gyrus, from baseline (T0) to post-intervention assessment at 6 months (T1). Increased resting-state fMRI activation in the left medial temporal region following recitation training is interpreted as reflecting enhanced cognitive reserve — the brain’s capacity to sustain normal cognitive function despite underlying neurodegenerative burden — consistent with the compensatory scaffolding framework of cognitive aging (Reuter-Lorenz and Park, 2014). We anticipate that the intervention group will demonstrate significantly greater increase in left medial temporal region activation compared to controls, with the effect expected to be more pronounced in participants with lower educational attainment — consistent with a compensatory neural response in individuals with lower a priori cognitive reserve (Stern et al., 2020).

The anticipated secondary outcome is intervention-induced change in task-based fMRI activation within bilateral fronto-striatal regions during active recitation of Chinese classics from T0 to T1. The fronto-striatal network — encompassing the dorsolateral prefrontal cortex, anterior cingulate cortex, caudate nucleus, and putamen — supports executive control, phonological working memory, and procedural learning, all of which are recruited during complex verbal recitation tasks (Ranjan and Singh, 2025). A training-induced decrease in fronto-striatal activation during recitation is the anticipated direction of effect, interpreted as increased neural efficiency: as recitation becomes more automatized through practice, the same cognitive output is achieved with lower metabolic demand, analogous to skill-learning-related neural efficiency observed in other trained cognitive domains (Kelly and Garavan, 2005).

EEG parameters and gut microbiota/metabolomic composition are designated as exploratory outcomes. Given that the study population comprises individuals with SCD, rather than overt neurodegenerative disease, significant alterations in EEG parameters may not be observed. EEG is included as an exploratory measure to characterize the electrophysiological signature of the SCD sample at baseline, and to detect any subclinical signal changes that may warrant inclusion as a sensitive biomarker in a future confirmatory trial. For the microbiota profile, the recitation training group is expected to show increased gut microbial alpha-diversity and enrichment of beneficial genera, including *Prevotella*, *Faecalibacterium*, and *Megamonas* (Sun Y. et al., 2023), as well as increased abundance of short-chain fatty acid-producing taxa, such as *Ruminococcus*, *Roseburia*, *Subdoligranulum*, and *Bifidobacterium* (Swarup et al., 2025), compared to controls. Results will be reported descriptively, including individual-level variability data, and effect sizes with 95% confidence intervals will be provided to inform the design of a future confirmatory trial.
